# Characteristics of *Streptococcus pneumoniae* colonising Cypriot children during a period of high PCV10 and PCV13 vaccine coverage: serotypes and antimicrobial resistance

**DOI:** 10.1186/s12879-026-13924-0

**Published:** 2026-07-04

**Authors:** Gavriella Ioannou Vassiliadou, Petros Ladas, Michail Mendris, Stella Mazeri, Maria Michail, Nelly Paphiti, Evi Eracleous, Paris Iacovides, Christos Economou, Charalampos Hadjigeorgiou, Kyriaki Tsokkou, Stelios Thomaides, Panagiota Skapoullarou, Michalis Iasonidis, Elli Siama, Lamprini Markaki, Iliada Evripidou, Athanasios Michos, Georgios Nikolopoulos, Maria Tsolia, Maria Koliou Mazeri

**Affiliations:** 1 University of Cyprus Medical School, Nicosia, Cyprus; 2 Limassol General Hospital, Limassol, Cyprus; 3 The Roslin Institute, Royal (Dick) School of Veterinary Studies, University of Edinburgh, Easter Bush, Midlothian, UK; 4 Private Pediatric Practice, Nicosia, Cyprus; 5 Private Pediatric Practice, Larnaca, Cyprus; 6 Private Pediatric Practice, Famagusta, Cyprus; 7 Private Pediatric Practice, Limassol, Cyprus; 8 Private Pediatric Practice, Paphos, Cyprus; 9 First Department of Pediatrics, Infectious Diseases and Chemotherapy Research Laboratory, Medical School, National and Kapodistrian University of Athens, ’Aghia Sophia’ Children’s Hospital, Athens, Greece; 10 Second Department of Pediatrics, National and Kapodistrian University of Athens, “A&P Kyriakou” Children’s Hospital, Athens, Greece; 11 Department of Paediatrics, Archbishop Makarios III Hospital, Nicosia, Cyprus

**Keywords:** *Streptococcus pneumoniae*, Colonisation, Antimicrobial resistance, Pneumococcal conjugate vaccines

## Abstract

**Background:**

Nasopharyngeal carriage of *Streptococcus pneumoniae* is a key factor for transmission, invasive disease, and antimicrobial resistance. Data on pneumococcal carriage in children in Cypriot are limited.

**Methods:**

We conducted a cross sectional study from December 2023 to May 2024, during a period of high PCV10 and PCV13 vaccine coverage in Cyprus, including children aged six months to six years who attended outpatient clinics in Cyprus. Nasopharyngeal swabs were cultured, and *Streptococcus pneumoniae* isolates were identified using conventional methods and confirmed by polymerase chain reaction. Serotyping was performed using multiplex polymerase chain reaction, latex agglutination, and whole-genome sequencing when needed. Antimicrobial susceptibility was assessed according to European guidelines. Logistic regression was used to identify risk factors for carriage.

**Results:**

A total of 809 children were included, 83.85% of whom were fully vaccinated with pneumococcal conjugate vaccines. The overall prevalence of carriage was 35.72%. Recent antibiotic use (OR 1.47, 95% CI 1.03–2.12) and daycare attendance (OR 1.89, 95% CI 1.37–2.63) were associated with increased carriage, whereas receipt of at least one vaccine dose was protective (OR 0.38, 95% CI 0.17–0.82). Among the 290 isolates, 34 distinct serotypes were identified, predominantly non-vaccine types including 15B, 23A, 23B, 24, 6C, and 15A. Most isolates (68%) were not covered by available vaccines. Resistance was highest for erythromycin (54.32%), tetracycline (37.28%), and clindamycin (31.65%). Multidrug resistance occurred in 28.94% of the isolates, mainly in serotypes 24, 15A, and 19A.

**Conclusions:**

Despite extensive vaccination, pneumococcal carriage in Cypriot children remains high, with predominance of non-vaccine serotypes and substantial antimicrobial resistance. Continuous surveillance is essential to guide vaccination and antimicrobial stewardship.

## Introduction

*Streptococcus pneumoniae* (pneumococcus) is a gram-positive diplococcus that was first identified in the late 19th century as the key causative agent of lobar pneumonia [1, 2]. It remains a major cause of illness and death in children worldwide and is the predominant pathogen isolated from community-acquired pneumonia, meningitis, and bacteremia [3]. The estimated mortality rate for invasive pneumococcal disease is approximately 15%, resulting in about 0.5 to 1 million deaths annually [3–5]. Children under five years of age, adults over 60, and immunocompromised individuals represent the groups with the highest risk [6]. Furthermore, pneumococcus is the leading cause of acute otitis media and the most frequent reason for antibiotic prescription in children, contributing significantly to antimicrobial resistance in the community [7].

*S. pneumoniae* frequently colonizes the nasopharynx, particularly in younger individuals [8]. Carriage plays a critical role in the transmission of bacteria within the community and the subsequent development of invasive diseases. Studies investigating pneumococcal carriage offer important insights into the distribution of strains, factors affecting colonization, and impact of vaccination programs. These findings also support the design of effective immunization strategies and monitoring of antimicrobial resistance [9, 10].

Recent data from the 2023 annual report of the European Antimicrobial Resistance Surveillance Network (EARS-Net) indicate an increasing trend in pneumococcal antibiotic resistance. Notably, 20.5% of invasive *S. pneumoniae* isolates were resistant to at least one antimicrobial agent (penicillin, third-generation cephalosporins, macrolides, and fluoroquinolones). The average rates of resistance to penicillin and macrolides across European countries were 15.5% and 17.8%, respectively [11].

In Cyprus, the 7-valent pneumococcal conjugate vaccine (PCV7) was introduced in the private sector in 2006. The 10-valent vaccine (PCV10) became publicly available and was incorporated into the national immunization program in 2010 and was fully subsidized by the government. Although PCV13 was introduced in 2011, it was not provided free of charge through the public health system until 2019. More recently, 15-valent (PCV15) and 20-valent (PCV20) vaccines were made publicly available free of charge in the early 2025.

Vaccination coverage with pneumococcal conjugate vaccines increased from 37.5% in 2009, during the PCV7 era, to 58.6% in 2012 following the introduction of PCV10 into the national immunization program among toddlers aged 17–24 months. The latest survey in 2019, when both PCV10 and PCV13 were in use, showed that coverage reached 73.3% [12].

Data on pneumococcal antimicrobial resistance from carriage studies in Cyprus are limited. Previous studies from 2007 to 2008 and 2013 to 2014 have described resistance and circulating serotypes during periods of low vaccine coverage [13, 14]. However, the current study reflects much higher vaccine coverage in children. This study aimed to identify the circulating serotypes and antimicrobial resistance of pneumococci colonizing the nasopharynx of children, considering the high vaccine coverage and prior to the universal implementation of the new PCV15 and PCV20 vaccines.

## Materials and methods

### Setting and study population

This was a cross sectional study investigating the characteristics of *S. pneumoniae* colonizing children in Cyprus between December 2023 and May 2024. The study population included healthy children or children with viral infections aged 6 months to 6 years. The participants were children who visited, along with their parents, either the outpatient clinics of a public hospital or the offices of private pediatricians.

The exclusion criteria were as follows: (i) receiving antibiotic therapy for any reason on the day of sampling, (ii) bacterial infection at the time of sampling, and (iii) if more than one participant was from the same household, the second participant was excluded. (iv) absence of written informed consent from parents or legal guardians. The sample size was based on the population of children aged six months to six years in the Republic of Cyprus. Previous studies have shown that approximately 30% of healthy children have *S. pneumoniae* [13, 14]. Using a 95% confidence level, 3.5% margin of error, and an estimated population of approximately 52,072 children in this age group, the required sample size was calculated as 651 participants. Assuming a response rate of 80%, the final estimated sample size was adjusted to approximately 815 children from all the districts.

Participant information was collected via a questionnaire completed by the parents or legal guardians, which included the following details: (i) the child’s sex, (ii) date of birth, (iii) district of residence, (iv) parents’ nationality, (v) history of breastfeeding, (vi) exposure to tobacco smoke, (vii) current viral respiratory infection, (viii) antibiotic use, (ix) presence of hypertrophic adenoids or tonsils, (x) history of adenoidectomy, (xi) day care attendance, (xii) underlying chronic conditions, and (xiii) vaccination status.

Nasopharyngeal samples were collected from the posterior nasopharynx by using sterile cotton swabs with a flexible wire (COPAN, Italy).

To protect participants’ personal data, participant information and nasopharyngeal samples were assigned a traceable code.

Fully vaccinated children were defined based on the official regimen recommended by the National Immunization Committee, which advises a 2 + 1 regimen for either the PCV10 or the PCV13 vaccine.

### Isolation of *S. pneumoniae* strains by culture

Collected samples were transported in transport medium and plated within 2 h onto blood agar plates enriched with 5% (v/v) defibrinated horse blood. The plates were incubated in a 5% CO₂ atmosphere at 35 °C for 24 h. Identification of *S. pneumoniae* strains were identified using classical methods based on colony morphology and optochin susceptibility [15].

### Antibiotic susceptibility testing

Susceptibility testing was performed on Mueller-Hinton agar plates supplemented with 5% (v/v) defibrinated horse blood. All strains were tested for susceptibility to oxacillin, erythromycin, clindamycin, trimethoprim/sulfamethoxazole, tetracycline, vancomycin, and linezolid using the antibiotic disk diffusion method. Additionally, the minimum inhibitory concentrations (MICs) for penicillin and cefotaxime were determined for all isolates using the E-test method and the corresponding MIC strips (Liofilchem, Teramo, Italy). MICs for other antibiotics were also assessed if the strains were found to be nonsusceptible to disk diffusion. The interpretation of *S. pneumoniae* susceptibility results was based on the EUCAST 2022 guidelines [16].

### DNA extraction of *S. pneumoniae*

Bacterial colonies identified as *S. pneumoniae* by colony morphology and optochin susceptibility was harvested in ddH_2_O. DNA extraction was performed using the Viasure DNA/RNA Pathogen Extraction Kit (Certest Biotec S.L. Spain), according to the manufacturer’s protocol. DNA extraction was performed using the KingFisher DUO system (Thermo Fisher Scientific Inc., USA).

### Serotyping and classification workflow

Serotyping of *Streptococcus pneumoniae* isolates was performed using a stepwise algorithm to ensure accurate and comprehensive strain characterization. Initially, multiplex real-time PCR assays were applied for serotype and serogroup identification, targeting the most common circulating pneumococcal serotypes. In cases where PCR results provided serogroup-level resolution or where additional confirmation was required, latex agglutination was performed using type- and group-specific antisera to further refine serotype identification. For isolates that remained non-typeable or required higher-resolution analysis, whole-genome sequencing (WGS) was applied as a reference method. WGS-based serotype prediction was performed using bioinformatic analysis of assembled genomes. Final serotype assignment was based on concordant results between methods, with WGS considered the highest-resolution method when discrepancies occurred. This hierarchical approach ensured robust serotype determination across all isolates.

### Multiplex real-time PCR for identification and serotyping of *S. pneumoniae*

PCR identification and serotyping assays were performed on cultured pneumococcal isolates obtained after microbiological culture of nasopharyngeal samples and not directly on clinical specimens. Identification of *S. pneumoniae* was performed by detecting *S. pneumoniae* genes lytA and piaB, as described in other studies [17, 18]. Identification assays were carried out as follows: (i) 20 µl total reaction, (ii) 10 µl of NZYSupreme Multiplex qPCR Probe Master Mix (2x), (iii) 1 µl of lytA primer/probe mix, (iv) 1 µl of piaB primer/probe mix, (v) 5 µl of extracted DNA, and (vi) 3 µl of ddH_2_O.

Serotyping of *S. pneumoniae* using Real-Time PCR identified 19 serotypes and 14 serogroups. Primers and probes for the assays have been best described in other studies. Pimenta et al. (2010) [19], Azzari et al. (2012) [20], Sakai et al. (2015) [21], and Messaoudi et al. (2016) [22]. Each serotyping assay was carried out in a triplex reaction as follows: (i) 20 µl total reaction, (ii) 10 µl of NZYSupreme Multiplex qPCR Probe Master Mix (2x), (iii) 1 µl of FAM primer/probe mix, (iv) 1 µl of JOE/HEX primer/probe mix, (v) 1 µl of Cy5 primer/probe mix, (vi) 5 µl of extracted DNA, and (vii) 2 µl of ddH_2_O.

Multiplex Real-Time PCR for identification and serotyping was performed using a Rotor Gene Q system (QIAGEN, Germany). The cycling conditions were as follows: 5 min at 95 °C, followed by 40 cycles of 5 s at 95 °C and 60 s at 60 °C.

### Agglutination method

Serogroups were confirmed, and final serotypes were identified using the slide agglutination method for *S. pneumoniae*, following protocol BRD0095/12–21 from the Bacteriology Reference Department of UK Health Security Agency, England. The method used group- and type-specific antisera obtained from Statens Serum Institute in Copenhagen, Denmark.

### Next generation sequencing

Library preparation of the *S. pneumoniae* samples was performed using the NexteraXT DNA library preparation kit (Illumina, UK). Starting DNA concentrations for samples were normalized to 1ng of DNA in 5 µl of DNase/RNase-free water. Normalization of samples was achieved by prior DNA fluorometric quantification of samples using a Qubit 1X dsDNA High Sensitivity Kit (Thermo Fisher Scientific, USA). Library preparation was performed in accordance with the manufacturer’s instructions. The generated library was sequenced on a NextSeq2000 (Illumina, UK) system using a NextSeq2000 P1 reagent kit (300 cycles) (Illumina, UK).

Analysis of the sequenced data was performed using a Linux system (Ubuntu 22.04.5 LTS). Quality control of reads was performed with fastqc (v0.12.1) and multiqc (v1.29) and read filtering with fastp (v1.0.1). The classification of reads was established using kraken2 (v2.14) and Bracken (v3.1). De novo assembly of reads was performed using Skesa (v2.5.1) [23]. The assembled reads were serotyped using SeroBA (v1.0.2) [24].

### Statistical analysis

Data were entered into a secure database and analyzed using the R software (version 4.4.2) [25]. Descriptive statistics were used to present the study’s main findings. All graphs were generated using the *ggplot2* package [26].

## Results

### Demographic characteristics, variables, and carriage prevalence

A total of 809 nasopharyngeal samples was collected for this study. The demographic characteristics of the children and related variables are summarized in Table 1. The mean age was 28.99 months (approximately 2.5 years). The geographic distribution of the sample reflected the general pediatric population, with 39.31% of the samples originating from Nicosia, 31.27% from Limassol, 16.44% from Larnaca, 6.55% from Famagusta, and 6.43% from Paphos.

Among children for whom vaccination data were available, 83.85% were fully vaccinated for their age, 12.15% were partially vaccinated, and 3.88% were unvaccinated according to the national immunization schedule. In addition, a subset of children had received an additional dose of PCV15 vaccine administered outside the national program; however, due to the heterogeneity and limited number of these cases, PCV15 was not analysed as a separate vaccination category.Overall, 35.72% of the participating children were found to be colonized by *S. pneumoniae*.

**Table 1:** Demographic characteristics of the children and the related variables

Variables	Categories	Frequency (%)	Colonization (%)
Gender	Male	408 (50.43)	143 (35.05)
	Female	401 (49.57)	146 (36.41)
Age^5^	< 12 months	160 (19.78)	53 (33.12)
	12–24 months	228 (28.18)	84 (36.84)
	25–36 months	166 (20.52)	62 (37.36)
	37–48 months	94 (11.62)	38 (40.43)
	49–60 months	100 (12.36)	34 (34)
	61–72 months	59 (7.29)	18 (30.51)
Ethnicity	Greek – Cypriot	713 (89.57)	254 (35.62)
	Other	83 (10.43)	290 (34.94)
Breastfeeding^1^	Yes	605 (75.25)	214 (35.37)
	No	199 (24.75)	73 (36.68)
Tobacco smoke exposure	Yes	378 (46.96)	139 (36.77)
	No	427 (53.04)	149 (34.89)
Health status at sampling	Healthy	456 (57.36)	144 (31.58)
	Viral infection	282 (35.47)	121 (42.91)
	Other	57 (7.17)	18 (31.58)
Antibiotic exposure^2^	Yes	181 (22.04)	81 (44.75)
	No	627 (77.60)	208 (33.17)
Daycare attendance^3^	Yes	488 (60.47)	206 (42.21)
	No	319 (39.53)	83 (26.02)
Siblings	0	352 (43.73)	115 (32.67)
	1	348 (43.23)	131 (37.64)
	≥ 2	105 (13.04)	42 (40)
Vaccination status	No vaccine	30 (3.88)	18 (60)
	Fully vaccinated with PCV10	146 (18.86)	53 (36.30)
	Fully vaccinated with PCV13	311 (40.18)	103 (33.12)
	Fully vaccinated with an additional dose^4^	192 (24.81)	60 (31.25)
	Partially vaccinated with PCV10	15 (1.94)	9 (60)
	Partially vaccinated with PCV13	79 (10.21)	32 (40.51)

^1^Includes children who were breastfed for at least 2 weeks, regardless of the total duration or whether breastfeeding was exclusive

^2^ Recent antibiotic use within the past 90 days

^3^Includes children attending daycare or supervised by an adult with at least two other children

^4^Children who have received an additional dose of PCV10, PCV13, or PCV15

^5^Two participants had missing age category information and were excluded from age-stratified analysis

### Risk factors for pneumococcal colonization

Results of the univariable analysis are presented in Table 2. No significant associations with pneumococcal carriage were found for age, sex, breastfeeding, tobacco smoke exposure or the presence of siblings.

**Table 2:** Univariable logistic regression of the association between potential risk factors and pneumococcal colonisation

Variables	Categories	Odds Ratio	CI 95%	*p*-value
Gender	Female	Ref	Ref	0.98
	Male	1.00	0.74–1.36	
Age	< 12 months	Ref	Ref	0.87
	12–24 months	1.17	0.76–1.83	
	25–36 months	1.12	0.70–1.82	
	37–48 months	1.31	0.75–2.27	
	49–60 months	1.04	0.59–1.82	
	61–72 months	0.86	0.42–1.70	
Breastfeeding	No	Ref	Ref	0.51
	Yes	0.89	0.63–1.26	
Tobacco smoke exposure	No	Ref	Ref	0.83
	Yes	1.03	0.76–1.40	
Health status at sampling	Healthy	Ref	Ref	0.02
	Viral infection	1.53	1.11–2.10	
	Other	0.90	0.46–1.67	
Antibiotic exposure	No	Ref	Ref	0.01
	Yes	1.66	1.17–2.36	
Daycare attendance	No	Ref	Ref	< 0.001
	Yes	1.98	1.44–2.74	
Siblings	0	Ref	Ref	0.26
	1	1.30	0.94–1.79	
	≥ 2	1.26	0.78–2.04	
Vaccination^1^	No	Ref	Ref	0.02
	Yes	0.40	0.18–0.85	

^1^Includes children who had received at least one vaccine dose

The results of the multivariable analysis are presented in Table 3. Recent antibiotic use within the past 90 days (OR: 1.47, 95% CI: 1.03–2.12) and attendance at daycare or care by an adult responsible for at least two other children (OR: 1.89, 95% CI: 1.37–2.63) were significantly associated with increased odds of carriage. In contrast, receipt of at least one vaccine dose was associated with reduced odds of colonization compared with unvaccinated children (OR: 0.38, 95% CI: 0.17–0.82).

**Table 3:** Final multivariable logistic regression model of the association between potential risk factors and pneumococcal colonisation

Variables	Categories	Odds Ratio	CI 95%	*p*-value
Antibiotic exposure	No	Ref	Ref	0.04
	Yes	1.47	1.03–2.12	
Daycare attendance	No	Ref	Ref	< 0.001
	Yes	1.89	1.37–2.63	
Vaccination^1^	No	Ref	Ref	0.01
	Yes	0.38	0.17–0.82	

^1^Includes children who had received at least one vaccine dose

### Prevalence and distribution of serotypes

**Fig. 1:**
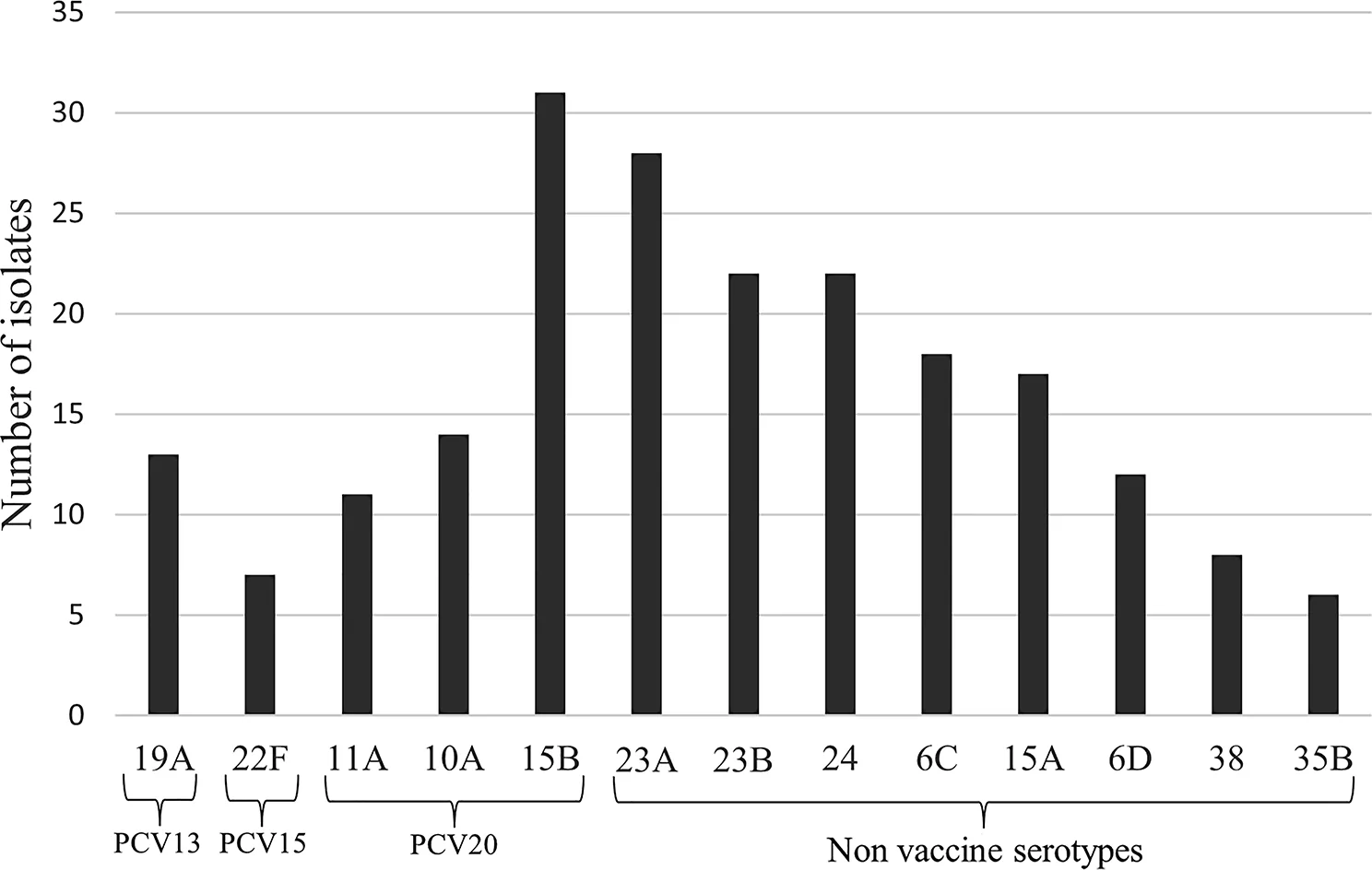
Distribution of the most frequent serotypes identified in pneumococcal carriers

A total of 290 *S. pneumoniae* isolates were identified. Serotype identification was successfully performed for 288 isolates, while two isolates could not be recovered for further analysis. Serotyping was performed using PCR (*n* = 243), latex agglutination (*n* = 201), and/or next-generation sequencing (NGS; *n* = 97), and some isolates were analysed using more than one method. Among the analysed isolates, serotype determination was not feasible for 19 despite the use of multiple methods. In total, 34 distinct serotypes were identified. Figure 1 presents the distribution of the serotypes identified using PCR, latex agglutination, and next-generation sequencing. The most common serotypes colonizing children were 15B (*n* = 31, 10.73%), 23A (*n* = 28, 9.69%), 23B (*n* = 22, 7.61%), 24 (*n* = 22, 7.61%), 6C (*n* = 18, 6.23%), 15A (*n* = 17, 5.88%), 10A (*n* = 14, 4.84%), 19A (*n* = 13, 4.50%), 6D (*n* = 12, 4.15%), 11A (*n* = 11, 3.81%), 38 (*n* = 8, 2.77%), 22F (*n* = 7, 2.42%), and 35B (*n* = 6, 2.08%).

Four serotypes of PCV13 (3, 14, 6A, and 19A) were identified among the isolates, with 19A being the most frequent. Overall, 68% of the isolates belonged to serotypes that were not covered by any of the conjugate vaccines, including PCV20.

### Antimicrobial resistance

The antimicrobial resistance patterns of the isolates against the tested antibacterial agents are summarized in Table 4. No resistance to linezolid or vancomycin was detected in any isolate. Overall, 51.84% of the isolates were fully susceptible to penicillin and only 1.10% were resistant to penicillin. A substantial percentage (91.76%) of isolates were fully susceptible to cefotaxime. The highest resistance rate was observed for erythromycin (54.32%), followed by tetracycline (37.28%), clindamycin (31.65%), and trimethoprim/sulfamethoxazole (24.33%). Moreover, 28.94% of *S. pneumoniae* isolates exhibited resistance to three or more antimicrobial classes.

**Table 4:** Antimicrobial resistance of the isolated strains

Antibiotic	Number of isolates (%)		
	Susceptible	Susceptible, increased exposure	Resistant
Penicillin	141 (51.84)	128 (47.06)	3 (1.10)
Cefotaxime	256 (91.76)	21 (7.53)	2 (0.71)
Erythromycin	127 (45.68)	-	151 (54.32)
Clindamycin	184 (66.19)	-	88^1^ (31.65)
TMP-SMX^2^	199 (75.67)	0 (0.00)	64 (24.33)
Vancomycin	279 (100)	-	0 (0.00)
Linezolid	279 (100)	-	0 (0.00)
Tetracycline	175 (62.72)	-	104 (37.28)

^1^7 of the isolates showed inducible resistance

^2^Trimethoprim/Sulfamethoxazole

Antimicrobial resistance patterns of the predominant carriage serotypes are shown in Table 5. Several serotypes exhibited high resistance to penicillin, erythromycin, and tetracycline. Multidrug resistance was observed in 61.90% of serotype 24 isolates, in 52.94% of serotype 15A isolates and 46.15% in both 10A and 19A.

**Table 5:** Antimicrobial resistance of the predominant serotypes

Antimicrobial resistance	Serotypes							
	15Β (*n* = 32)	23A (*n* = 28)	23B (*n* = 22)	24 (*n* = 22)	6C (*n* = 18)	15A (*n* = 17)	10A (*n* = 14)	19A^3^ (*n* = 13)
Non-susceptible^1^ to penicillin (%)	0.00	0.00	0.00	0.00	0.00	0.00	0.00	7.69
Non-susceptible^1^ to erythromycin (%)	67.74	57.69	13.64	76.19	66.67	82.35	53.85	46.15
Non-susceptible^1^ to tetracycline (%)	22.58	34.62	4.55	76.19	55.56	52.94	46.15	46.15
Multidrug resistance^2^	23.33	34.62	0.00	61.90	44.44	52.94	46.15	46.15

^1^“Non-susceptible” was defined as isolates categorized as “I” (susceptible, increased exposure) or “R” (resistant) according to EUCAST 2022 interpretive criteria

^2^Multidrug resistance: resistance to three or more classes of antimicrobials

^3^Serotype included in PCV13, PCV15, and PCV20

## Discussion

In this prospective study, we investigated the characteristics of *S. pneumoniae* colonizing children aged 6 months to 6 years during a period of high vaccination coverage of PCV10 and PCV13 in Cyprus. The overall pneumococcal carriage rate was 35.72%, which is consistent with findings from recent studies that reported rates between 25.4% and 37.7% [27–29]. Some studies have reported lower carriage rates, ranging from 9.8% to 17.8%, which is been attributed to high pneumococcal conjugate vaccine coverage in the studied populations [30, 31]. In contrast, higher carriage rates (40.1–48.6%) have been reported in studies using molecular detection methods applied directly to nasopharyngeal specimens, which generally have higher sensitivity compared with culture-based approaches [27, 32–34]. When compared with data from an earlier study conducted in Cyprus before the introduction of PCVs into the national immunization program, the carriage rate appears to have remained relatively stable [14].To investigate the association between potential risk factors and *S. pneumoniae* carriage, both univariable and multivariable analyses were performed. No statistically significant associations were identified with factors previously reported in the literature, such as age, sex, exposure to tobacco smoke, health status at the time of sampling, or presence of siblings [35, 36]. Regarding vaccination, receipt of at least one dose of any PCV appeared to reduce the likelihood of colonization, as vaccinated children were found to have a 62% lower risk of carriage than unvaccinated children. This protective effect is consistent with several previous studies that reported reduced pneumococcal carriage among PCV-immunized children. However, this association has not been consistently supported across all studies, with some investigations reporting similar carriage rates before and after the introduction of conjugate vaccines [37]. Recent antibiotic use within the past 90 days was significantly associated with an increased odds of pneumococcal carriage. This finding aligns with previous studies indicating that recent antibiotic exposure can disrupt nasopharyngeal microbiota, potentially facilitating colonization by *S. pneumoniae*. For instance, Lee et al. (2017) reported that recent antibiotic use was associated with a higher risk of colonization, predominantly serotype 19A, possibly reflecting the selection of resistant strains [38].

In the present study, attendance at day care or care by an adult responsible for at least two other children emerged as a significant risk factor, being associated with a nearly two-fold increase in the odds of *S. pneumoniae* carriage. According to the model proposed by Huagan and Finkelstein, day care attendance may account for 4%–56% of the variability in pneumococcal carriage prevalence across communities, with children attending day care having a two- to threefold higher likelihood of colonization compared with those who do not attend. Furthermore, previous studies have suggested that the duration of day care attendance influences the risk of carriage independent of the number of children enrolled, likely due to increased contact with colonized children or those with mild pneumococcal infections of the respiratory tract [39]. Further analysis of the data is warranted to adjust for all relevant variables and to draw more robust conclusions.

The use of multiple complementary methods for pneumococcal serotyping has strengthened the accuracy of our findings. Initial identification was performed using RT-PCR, which offers high sensitivity and specificity but limited ability to distinguish between certain serotypes that belong to the same serogroup, such as 6C/D and 15A/B. To refine the results and confirm these serogroups, latex agglutination was performed. Although it is a rapid and practical technique, its accuracy may be affected by subjective interpretation. The combined use of PCR and latex agglutination analysis proved to be a reliable approach for routine serotyping. In isolates for which the serotype could not be determined by either method, Next Generation sequencing (NGS) was employed, providing comprehensive and highly accurate results. Despite its high cost and the need for specialized bioinformatics support, NGS remains a valuable tool for resolving inconclusive cases and enhancing the robustness of pneumococcal characterization [40, 41].

Regardless of vaccination status, serotypes colonizing children were 15B, 23A, 23B, 24, 6C, 15A, 10A, 19A, 6D, 11A, 38, 22F, and 35B. Common colonizing serotypes, such as 15B, 19A, 11A, and 10A, have also been reported in other countries of Southeastern Europe and the Eastern Mediterranean region, including Greece, Turkey, and Serbia, according to recent carriage studies [31, 32, 42]. In contrast, data from South Asia showed a different serotype distribution, suggesting geographic variability in the epidemiology of *S. pneumoniae* [29, 43]. This marked geographical diversity in serotype distribution may reflect underlying socioeconomic differences between countries as well as variations in pneumococcal vaccination policies and schedules.

Several serotypes identified in the present study have been associated with invasive pneumococcal disease (IPD). According to data from the 2018 annual epidemiological report of the ECDC and a 2022 retrospective study including 30 high-income countries, serotypes 19A, 22F, 10A, and 11A were among the most frequently isolated serotypes from patients with invasive disease [44, 45]. The same serotypes were detected in our study (Fig. 1), raising concern about their potential clinical relevance.

Children were classified into different vaccination categories based on the national immunization schedule for PCVs in Cyprus and their age. Between 2011 and 2019 the PCV10 was the vaccine offered free to children, while from 2019 until today, they switched to PCV13. In 2019, the national coverage of PCVs was estimated at 73.30% [12]. In the present study, 83.85% of the children were fully vaccinated with PCV10 or PCV13, 12.15% were partially vaccinated, and 3.88% were unvaccinated. In comparison, vaccination coverage among children across Europe ranges from 30% to 80% [46]. Increasing vaccination rates and ensuring the completion of the full vaccination schedule are essential to strengthen herd immunity and limit the circulation of invasive *S. pneumoniae* strains.

The distribution of circulating *S. pneumoniae* serotypes is a dynamic process that changes over time, primarily due to the replacement of vaccine-covered serotypes by non-vaccine types. In recent years, with the introduction of new PCVs and improvements in serotype surveillance and detection, this phenomenon has become more pronounced. Interestingly, serotypes that have emerged to replace vaccine-covered ones often exhibit lower pathogenic potential [47, 48].

In Cyprus, two previous carriage studies compared serotype distribution across three distinct periods: 2007–2008 (low PCV7 coverage), 2013–2014 (following the introduction of PCV10 and PCV13), and the present period, just before the introduction of PCV15 and PCV20 (2023–2024). During the first period, the predominant serotypes were 15B, 6B, 23A, 23B, 19F, 15C, and 7B, with 6B and 19F being included in PCV7 [49]. During the second period, the most frequent serotypes were 23A, 23B, 15A, 15B, 6C, 19A, and 35F, with 19A being covered by PCV13. In the present study, serotypes 15B, 23A, 23B, 6C, and 15A continued to circulate, remaining outside the coverage of PCV10 and PCV13 [13]. Nonetheless, serotype 19A persists at a stable rate, while others, such as 24 and 6D, previously uncommon, now appear with increased frequency. Most importantly, a total of 68% of the isolates belonged to serotypes not included in the currently available PCVs, including 23A, 23B, and 24.

Serotype 19A was among the most frequent serotypes detected in both fully PCV10- and PCV13-vaccinated children, which is consistent with findings from a previous carriage study in Cyprus [13]. Observational studies have shown that the introduction of PCV7 and PCV10 was followed by an increase or no change in 19A carriage, whereas PCV13 use led to a decline in this serotype, though not completely [50–52]. PCV13 induces an antibody response against 19A, enhancing both direct and herd protection [53].

In this study, serotype 19A exhibited reduced susceptibility to penicillin, moderate resistance to erythromycin, and one of the highest rates of multidrug resistance among the detected serotypes (Table 4). Serotype 19A is associated with invasive disease, demonstrates high levels of multidrug antimicrobial resistance, and has the ability to persist in carriage regardless of the pneumococcal conjugate vaccine (PCV) type, which highlights the potential need for improved vaccination strategies capable of eliciting stronger neutralizing antibody responses against this serotype.

Serotype 15B was the most frequently detected serotype in the present study. While it exhibited low rates of non-susceptibility to penicillin, its high resistance to erythromycin is concerning, and a moderate level of multidrug resistance indicates the presence of strains resistant to multiple antibiotic classes (Table 4). Serotype 15B is also a recognized cause of otitis media, and has been linked to severe infections, including meningitis, as well as higher mortality rates [54]. The combination of high prevalence and resistance underscores the importance of targeting 15B in vaccine development.

In the present study, important findings were revealed regarding *S. pneumoniae* antimicrobial resistance, which is consistent with the 2023 EARS-Net report, where increasing resistance to several antimicrobial agents has been documented. Although full resistance was not detected with critical antibiotics, such as penicillin, cefotaxime, linezolid, and vancomycin, a considerable level of resistance was observed in other classes, particularly erythromycin, tetracycline, clindamycin, and trimethoprim/sulfamethoxazole. Given the high resistance rates, erythromycin and clindamycin may not be suitable for empirical monotherapy for pneumococcal infections. These findings are in line with previous carriage studies conducted in Cyprus, although erythromycin resistance appears to have increased compared with earlier data [55]. In addition, a higher proportion of isolates in this study were classified as penicillin susceptible with increased exposure, suggesting that high-dose penicillin regimens should continue to be recommended for the treatment of non-meningitis pneumococcal infections. The finding that 28.94% of the isolates were multidrug-resistant (resistant to three or more antibiotic classes) is consistent with the results of several studies and underscores the urgent need to strengthen antimicrobial stewardship measures. When resistance patterns were analyzed by serotype, higher rates of non-susceptibility to erythromycin and multidrug resistance were observed among the most prevalent serotypes. Importantly, serotypes 24 and 15A, which are not included in the currently available vaccines, exhibit substantial multidrug resistance.

The prospective design of this study has several limitations. First, participating pediatricians were selected based on their willingness to participate, which may have introduced selection bias and resulted in a non-random sample. Although most participants were part of the national General Healthcare System and children from all districts of Cyprus were included, supporting the representativeness of the sample, the possibility of selection bias cannot be excluded. Second, the cross-sectional nature of the study limits the ability to establish causal relationships between identified risk factors and pneumococcal carriage. In addition, although multivariable analysis was performed, residual confounding from unmeasured factors cannot be ruled out. Third, the study period did not cover all seasons, and therefore potential seasonal variations in pneumococcal carriage could not be assessed. Fourth, despite the use of multiple complementary methods for serotype identification, a small number of isolates remained non-typeable, which may have slightly affected the accuracy of serotype distribution estimates. In addition, no cases of multiple-serotype carriage were identified. However, because serotyping was performed on cultured isolates and no molecular methods specifically designed to detect co-colonization were applied directly to nasopharyngeal specimens, carriage of additional serotypes present at low abundance cannot be completely excluded. Fifth, although whole-genome sequencing (WGS) was used for serotype identification in selected isolates, detailed genomic analyses such as multilocus sequence typing (MLST), phylogenetic analysis, and investigation of potential serotype switching were beyond the scope of the present study. Therefore, clonal relationships and pneumococcal population dynamics could not be assessed. Finally, MIC determination using gradient diffusion methods (E-test) for penicillin and cefotaxime may be associated with minor variability compared with reference broth microdilution methods and could potentially lead to slight underestimation of MIC values, as highlighted in EUCAST recommendations. Nevertheless, this study provides up-to-date data on *Streptococcus pneumoniae* carriage and antimicrobial resistance in Cypriot children, contributing to national surveillance efforts.

## Conclusions

In conclusion, this study provides an updated overview of *S. pneumoniae* carriage in Cypriot children and represents the first investigation in Cyprus to integrate molecular techniques, including RT-PCR and whole-genome sequencing, with latex agglutination, offering a highly feasible and accurate approach for characterization of pneumococcal carriage to date. Detailed information on serotype distribution, factors influencing carriage, and indirect vaccine effects is essential for understanding pneumococcal dynamics. While overall carriage rates remained stable, there was a shift toward non-vaccine serotypes, particularly 23A, 23B, 24, 6C, and 15A, several of which exhibited notable antimicrobial resistance. High resistance to erythromycin and clindamycin limits their use as first-line empirical therapies, whereas the increased frequency of isolates requiring high-dose penicillin highlights the need for careful antibiotic selection. These findings underscore the importance of continuous surveillance of circulating serotypes and antimicrobial resistance together with efforts to optimize full vaccination coverage. Strengthening the national immunization program is likely to play an important role in limiting the spread of highly pathogenic and resistant strains and safeguard public health.

## Data Availability

The data supporting the findings of this study are not publicly available due to ethical and privacy restrictions; however, all relevant aggregated data are included within the article. Detailed data are available from the corresponding author upon reasonable request.

## Declarations

### Data access statement

The datasets generated and analysed during the current study are available in the European Nucleotide Archive repository under accession number [PRJEB111125].

## Abbreviations

PCV: Pneumococcal Conjugate Vaccine
PCV7: 7-valent Pneumococcal Conjugate Vaccine
PCV10: 10-valent Pneumococcal Conjugate Vaccine
PCV13: 13-valent Pneumococcal Conjugate Vaccine
PCV15: 15-valent Pneumococcal Conjugate Vaccine
PCV20: 20-valent Pneumococcal Conjugate Vaccine
*S. pneumoniae*: *Streptococcus pneumoniae*
NGS: Next-Generation Sequencing
RT-PCR: Real-Time Polymerase Chain Reaction
MIC: Minimum Inhibitory Concentration
EARS-Net: European Antimicrobial Resistance Surveillance Network
EUCAST: European Committee on Antimicrobial Susceptibility Testing
IPD: Invasive Pneumococcal Disease
OR: Odds Ratio
CI: Confidence Interval
